# AI chatbots as ‘pocket doctors’: intimate health support for young women in Lebanon

**DOI:** 10.1186/s12889-025-25386-1

**Published:** 2025-11-17

**Authors:** Anthony Mina, Elie Ghadban, Tigresse Boutros, Naya Saade, Lynn Fayad, Michel Abi Karam, Sabine Breidi, Marc Elias, Maryline Ghosh, Layane El Khoury, Nicolas Naassan, Raghid El Khoury

**Affiliations:** 1 School of Medicine and Medical Sciences, Holy Spirit University of Kaslik, Jounieh, Lebanon; 2 Urology Department, Notre Dame Des Secours University Hospital, 93, Byblos, 3 Lebanon; 3 Faculty of Medicine, American University of Beirut Medical Center, Beirut, Lebanon

## Abstract

**Background:**

In conservative societies such as Lebanon and the broader Middle East and North Africa region, gynecological and intimate health issues are heavily stigmatized, limiting young women’s access to care due to fear of judgment, privacy concerns, and cultural taboos. These barriers often result in delayed diagnoses and poorer health outcomes. Large Language Models, such as ChatGPT and Gemini, have emerged as digital tools offering anonymity, reduced embarrassment, and accessibility, potentially serving as discreet “pocket doctors” for sensitive health concerns. However, little is known about young women’s perceptions and use of artificial intelligence for intimate health topics in such contexts.

**Methods:**

A cross-sectional quantitative study surveyed 525 female university students in Lebanon (ages 18–35) to assess their use, perceptions, drivers, and barriers related to artificial intelligence chatbots for intimate and general health concerns.

**Results:**

The study included 525 young Lebanese women with a mean age of 22.44 ± 3.74 years. Regarding AI chatbot use, the most common intimate health topics included menstrual problems (43.8%) and polycystic ovary syndrome (33.3%), while physical fitness (59.8%) and mental health (48.8%) were the predominant general health topics. The primary barriers to chatbot use were concerns about accuracy (85.5%) and lack of physical examination (85.3%), while key motivators included saving time (71.0%) and avoiding embarrassment (43.4%). Younger women were more likely to use artificial intelligence tools to avoid judgment and cost. Cluster analysis revealed distinct user profiles, including a super-user group with intensive engagement across sensitive health domains.

**Conclusion:**

Large language models serve as accessible, non-judgmental digital confidants for young Lebanese women’s intimate health concerns, addressing socio-cultural stigma and healthcare system limitations. While promising, they should complement, not replace, professional care due to limitations in clinical reasoning, physical examination, and privacy concerns. Integrating artificial intelligence chatbots thoughtfully may enhance health information access and reduce barriers in stigmatized settings.

**Supplementary Information:**

The online version contains supplementary material available at 10.1186/s12889-025-25386-1.

## Introduction

Long before the advent of antibiotics to combat infections and anesthetics to alleviate pain, the practice of medicine was grounded in two fundamental elements: curiosity and care. These constituted the earliest tools of a physician: human connection and the persistent pursuit of understanding [1]. Over time, this curiosity merged with innovation, leading to the development of technologies that modified not only the management of disease but also broader conceptions of health. Nowadays, curiosity manifests in digital form through Large Language Models (LLMs) such as the Chat Generative Pre-Trained Transformer, or ChatGPT [2], a language model developed by OpenAI, and Gemini, developed by Google [3]. Although they do not hold a medical degree, these Artificial Intelligence (AI) chatbots have found their way into healthcare conversations and have been applied in fields like surgery, oncology, and orthopedics, revealing themselves to be versatile and widely useful, as is the example of ChatGPT [2, 4]. Unlike traditional digital tools such as Google search engines or social media platforms, which provide fragmented and sometimes non-validated health information, AI chatbots simulate interactive conversations [5]. This interactive, personalized format allows users to ask sensitive questions in real time and receive personalized responses in a confidential, judgment-free space [6, 7]. These features distinguish chatbots from other digital information sources and make them particularly relevant for intimate health concerns [6, 7].

In general, gynecological and intimate health issues are heavily stigmatized, making it difficult, especially for young, unmarried women, to seek care without fear of judgment or social consequences, notably in Lebanon and similar conservative societies such as the Middle East and North Africa region [8, 9]. This culture of secrecy leads to delays in diagnosis and treatment [10]. Fear of social judgment, concerns about privacy, and cultural taboos surrounding sexuality often discourage adolescents and young adults from seeking professional care [10]. This silence is reinforced by limited sexual health education [11] and a lack of youth-friendly, confidential services [6, 12], contributing to misinformation, delayed diagnoses, and poorer health outcomes [6, 10, 12]. In Lebanon specifically, sexual and reproductive health knowledge among unmarried women is particularly low, with only 8.8% demonstrating adequate awareness of contraception, premarital health screening, and sexually transmitted infections [12]. Strong cultural taboos, fear of pregnancy, and the absence of youth-friendly confidential services further discourage single women from seeking care, reinforcing silence and misinformation [12].

As a result, digital tools, including AI-driven chatbots, are increasingly explored as alternative sources of support for sensitive health concerns. Recent studies, including those by Marcantonio et al. and Esmaeilzadeh et al., underline the growing reliance on AI chatbots to address intimate health concerns that are difficult to raise in traditional care settings [6, 7]. These platforms offer particular promise in sensitive areas of health, including gynecological issues, where patients may hesitate to seek in-person care due to stigma or embarrassment. Supporting this potential, a recent study by Lin et al. found that, for example, ChatGPT outperformed human candidates in a virtual Objective Structured Clinical Examination in obstetrics and gynecology, demonstrating its ability to deliver accurate responses under clinical conditions [13]. Furthermore, among the key advantages are anonymity, reduced embarrassment, permanent uninterrupted accessibility and potential to serve as an initial triage tool [6, 7, 14]. Notably, similar dynamics were explored in a meta-analysis by Kim, which appraised the positive effects of chatbot interventions on women’s health outcomes, revealing high acceptability and practicality for these digital tools [15]. More broadly, studies on public attitudes regarding the clinical use of Artificial Intelligence (AI) indicate that patients, including women, tend to form positive perceptions of these technologies based on prior knowledge and familiarity, particularly when that familiarity is rooted in health-related contexts [16–18].

While these benefits seem convincing, they must be weighed against numerous yet-to-be explored limitations. In fact, despite outperforming human candidates in virtual clinical assessments [13], LLMs remain tools trained on probabilistic patterns, not medical reasoning [19, 20] and they lack diagnostic authority and cannot individualize care [20, 21]. Another concern when applied to healthcare is the lack of privacy, as suggested by Wang et al. [20], since it interacts directly with patients and gathers their personal details [22, 23], posing a fundamental flaw in addressing women’s health. Inability to conduct physical exams or interpret clinical findings further complicates matters [24] and can potentially lead to dreadful delays or inadequate delivery of care if users rely solely on AI chatbots such as ChatGPT rather than seeking medical attention [23, 24].

Despite the enthusiasm around the use of LLMs in healthcare, one critical question remains underexplored: how do young people, and particularly women, perceive their use for intimate health concerns? While a chatbot may seem like a non-judgmental “pocket doctor” [25], especially for stigmatized topics like gynecological health, little research has examined women’s comfort, trust, or willingness to rely on LLMs instead of physicians. This gap is particularly important given the rising use of digital tools for private medical queries [26, 27]. Yet gender-specific data, information on stigma, and insights into the tension between convenience and medical reliability remain scarce in the literature.

### The present study

Given the combined crises Lebanon has endured in recent years, marked by economic collapse, rising poverty, political instability, ongoing regional conflicts and the COVID-19 pandemic, in addition to the catastrophic Beirut explosion of 2020 [28, 29], it becomes vital to rethink how healthcare is accessed and perceived [30], especially in relation to intimate and stigmatized concerns. Such overlapping instabilities intensify psychological distress [30], and deepen public distrust toward the Lebanese government [31], and by extension, the healthcare sector. This distrust is further exacerbated by the country’s alarming medication shortage and the near-collapse of its healthcare system, revealing the extent of the latter’s chronic instability [28, 32]. Consequently, young people, particularly women, might find themselves even more reluctant to seek help for sensitive health issues, due to increased fear of privacy breaches, amplified stigma, and unaffordable costs [33]. In response to these complex barriers, LLMs could work as potentially empowering tools.

To the best of our knowledge, this is the first study to examine how young female individuals perceive and engage with LLMs for gynecological and intimate health concerns as “friendly pocket doctors”. The aim is to explore not only the potential uses of AI chatbots but also the perceptions that act as drivers, barriers, and reflections of broader attitudes toward seeking support for sensitive health issues through this technology. Ultimately, the goal is to determine whether such technology can bridge the gaps created by both socio-cultural stigma and specific challenges of the Lebanese healthcare system. To better understand these dynamics, this study is guided by the Technology Acceptance Model (TAM), a widely used framework for examining how people adopt new technologies in healthcare. According to TAM, individuals are more likely to embrace a technology when they believe it is useful and easy to use, as these perceptions shape their attitudes and intentions to engage with it. Recent applications of TAM to large language models highlight that, beyond usefulness and ease, factors such as perceived risk and social influence also play an important role in determining acceptance. Using TAM as a lens allows us to make sense of both the drivers, such as convenience and stigma reduction, and the barriers, such as concerns over accuracy, privacy, and the lack of physical examination, that influence how young women view chatbots for intimate health concerns [34, 35].

## Methods

### Ethical approval

Ethical clearance was granted by the Ethics Committee of Notre Dame des Secours University Hospital (NDS-UH) on February 6, 2025 (Reference: CR1/2025). All research activities were conducted in accordance with institutional standards. Particular attention was given to data privacy and digital security, given the sensitivity of intimate health topics and the involvement of AI-driven tools.

### Study design and participants

A cross-sectional quantitative study was conducted to assess the perceptions, drivers, and barriers related to the use of AI chatbots for sensitive health concerns among young female university students in Lebanon. Eligible participants were women aged 18–35 years, currently enrolled in universities across Lebanon, regardless of their field of study. Recruitment targeted a diverse sample to involve a broad range of experiences and attitudes, including both health-related and non-health-related academic backgrounds. Participation was voluntary and anonymous, with informed consent obtained electronically prior to data collection.

### Minimal sample size calculation

The minimum sample size was determined to ensure sufficient statistical power for detecting meaningful associations in both bivariate and multivariable analyses. Assuming a moderate effect size (Cohen’s d = 0.3), a 95% confidence level, and 80% power, the required sample was estimated at 350 participants. The final sample included 525 respondents, exceeding the minimum threshold and allowing for effective analysis [36].

### Questionnaire and data collection

The self-administered questionnaire was designed to be completed in approximately ten minutes and was divided into two main sections. Data was collected online using Google Forms survey between February and May 2025. The survey link was disseminated via university mailing lists, student groups, and peer networks using a “snowball” sampling approach. All responses were anonymous and stored in password-protected files accessible only to the research team.

Digital consent was required to access the survey, and no identifiable personal data were collected.

#### Section 1: sociodemographic and health characteristics

The first section gathered socio-demographic and academic information, including age, university affiliation, field of study (health-related or non-health-related), and region of residence. Additional questions assessed basic health status, such as chronic medication use and smoking status.

#### Section 2: chatbot Use, Perceptions, and attitudes

The second section focused on participants’ experiences and attitudes regarding the use of AI chatbots for health-related concerns. This included items on:

- Utilization of AI chatbots (e.g., ChatGPT, Gemini) for a range of general and intimate health topics, such as menstrual problems, polycystic ovary syndrome (PCOS), vaginal discharge, mental health, physical fitness, and nutrition.
- Knowledge and perceptions of AI chatbots, including perceived reliability and trustworthiness.
- Drivers for consulting AI chatbots (e.g., saving money, avoiding embarrassment, fear of judgment, convenience).
- Barriers to chatbot use (e.g., concerns about accuracy, privacy, lack of emotional support, need for physical examination).
- Broader attitudes toward technology and healthcare-seeking behaviors.

While no existing validated tool fully addressed the scope of our inquiry, the questionnaire items were constructed based on adaptation from literature regarding digital health and trends of AI chatbot use. Key references informed the item development, in order to ensure relevance and alignment with contemporary discourse in the field, especially regarding sexual and reproductive health and mental wellbeing [15, 37–39].

The questionnaire was pilot-tested among a small group of university students to ensure clarity and cultural appropriateness. In fact, to improve clarity and ensure face validity, the instrument was pilot tested with 18 female university students, aged 18–25 years, recruited from different faculties. Participants completed the draft questionnaire and provided structured feedback on the clarity, wording, and relevance of items. Descriptive analysis of pilot data confirmed acceptable variability across items, with no evidence of floor or ceiling effects. Based on participants’ feedback, adjustments were made: (1) rewording several items to improve clarity, (2) refining the response scale for consistency, and (3) removing two redundant questions. The revised version was then finalized and used in the main study. Data collection was conducted online, with responses securely stored to maintain participant confidentiality. The questionnaire was distributed via Google Forms, and digital consent was required prior to proceeding. If consent was not given, the form automatically exited.

### Statistical analysis

All statistical analyses were conducted using SPSS version 26. Descriptive statistics summarized participant characteristics and patterns of AI chatbot use. Normality of continuous variables was assessed using skewness and kurtosis. Independent samples t-tests and chi-square tests were used for bivariate analyses to compare age and health-related field status with chatbot use, drivers, and barriers, retaining variables with *p* < 0.20 for multivariable modeling. Multiple linear regression identified predictors of age, while logistic regression examined factors associated with being in a health-related field, with model fit assessed by R² and the Hosmer-Lemeshow test [40]. Multicollinearity was evaluated using variance inflation factors. Finally, K-means cluster analysis was performed on binary indicators of chatbot use across health domains to identify user profiles [41]. Statistical significance was set at *p* < 0.05.

## Results

### Descriptive analysis

The study included 525 young Lebanese women with a mean age of 22.44 ± 3.74 years. Just over half (55.8%) were engaged in health-related fields (Table 1). Most participants were single (65.1%), non-smokers (80.6%), and had private health coverage (73.9%). Participants’ self-reported trust in AI averaged 5.19 out of 10, while reliance on professionals remained higher (mean: 6.04), compared to AI tools (4.70), Google (4.92), or social media (4.38). Perceived financial burden averaged 4.91/10 (Table 2).

Tables are organized thematically to distinguish between health domains, attitudinal constructs, and analytic levels, ensuring clearer interpretation of patterns across chatbot usage, motivations, and user subgroups.

**Table 1:** Socio-Demographic characteristics

Variable	Category	Frequency (*n*)	Percentage (%)
Health-related Field	Yes	293	55.8
	No	232	44.2
Smoking	Yes	102	19.4
	No	423	80.6
Alcohol	Yes	212	40.4
	No	313	59.6
Marital Status	Single	342	65.1
	In a relationship	155	29.5
	Married	28	5.3
Health Coverage	Private	388	73.9
	Public	65	12.4
	Out-of-pocket	72	13.7
Chronic Medications	Yes	457	87.0
	No	68	13.0
First Choice for Medical Advice	Search engines like google	166	31.6
	LLMs (ChatGPT, Gemini etc.)	116	22.1
	Healthcare professional	233	44.1
	Social media platforms	10	1.9

**Table 2:** Key numerical variables

Variable	Mean	SD	Min	Max
Age	22.44	3.74	18	35
Financial burden (1–10)	4.91	2.16	1	10
Trust in AI (1–10)	5.19	1.99	1	10
Rely on AI (1–10)	4.70	2.19	1	10
Rely on Google (1–10)	4.92	2.23	1	10
Rely on social media (1–10)	4.38	2.08	1	10
Rely on professionals (1–10)	6.04	2.51	1	10

Regarding AI chatbot use for intimate health issues, menstrual problems (43.8%) and polycystic ovary syndrome (33.3%) were the most common topics (Table 3). For general health, physical fitness (59.8%) and mental health (48.8%) were predominant (Table 4). The main barriers to AI use were concerns about accuracy (85.5%) and lack of physical examination (85.3%), while the primary drivers included saving time (71.0%) and avoiding embarrassment (43.4%) (Tables 5 and 6). No individuals were excluded from this study and no missing data is reported.

**Table 3:** Use of AI for intimate health topics

Concern	*n* (Yes)	%
Menstrual problems	230	43.8
PCOS	175	33.3
Vaginal discharge/infections	119	22.7
UTI	111	21.1
Pelvic pain	105	20.0
STDs	80	15.2
Breast health	83	15.8

**Table 4:** Use of AI for general health topics

Topic	*n* (Yes)	%
Mental Health	256	48.8
Physical Fitness	314	59.8
Steroids	46	8.8
Supplements	112	21.3
Nutrition	303	57.7

**Table 5:** Barriers to AI chatbot use

Barrier	*n* (Yes)	%
Accuracy Concerns	449	85.5
Physical Exam Needed	448	85.3
Contextual Understanding Lacking	393	74.9
Privacy Risks	282	53.7
Lack of emotional support	212	40.4

**Table 6:** Drivers for using AI chatbots

Driver	*n* (Yes)	%
Save Time	373	71.0
Embarrassment	228	43.4
Save Money	214	40.8
Fear of Judgment	175	33.3
Lack of Access to Healthcare Providers	139	26.5

### Bivariate analysis

Age differences were significant for several health topics and motivational drivers. Younger participants were more likely to use AI chatbots for mental health (*p* = 0.036), physical fitness (*p* = 0.027), and menstrual problems (*p* = 0.050), whereas older participants more frequently consulted AI for pregnancy-related concerns (*p* = 0.008) (Table 7). Similarly, younger age was associated with using AI to save money (*p* = 0.025), avoid embarrassment (*p* = 0.004), and due to fear of being judged (*p* < 0.001) (Table 8).

**Table 7:** Age differences by health topics

Variable	Grouping Variable	Mean Difference	95% CI	t	df	*p* value
Age (years)	Mental health	0.684	[0.045–1.323]	2.102	523	**0.036**
	Physical Fitness	0.737	[0.086–1.388]	2.223	523	**0.027**
	Performance Enhancing Drugs	−0.046	[−1.181−1.088]	−0.08	523	0.936
	Fitness Supplements	−0.149	[−0.932−0.634]	−0.374	523	0.708
	Nutrition Plans	0.181	[−0.468−0.830]	0.547	523	0.584
	Menstrual Problems	0.645	[0.001–1.289]	1.967	523	**0.050**
	STDs	−0.621	[−1.52−0.270]	−1.369	523	**0.172**
	Infertility	−0.784	[−1.762−0.194]	−1.576	523	**0.116**
	Vaginal Discharge	0.553	[−0.211;1.318]	1.421	523	**0.156**
	Urinary Tract Infections	−0.064	[−0.849;0.722]	−0.160	523	0.873
	Pelvic Pain	0.212	[−0.590;1.014]	0.519	523	**0.604**
	Pain during intercourse	−0.783	[−0.1904;0.339]	−1.371	523	**0.171**
	Urinary Incontinence	−1.127	[−2.269;0.015]	−1.939	523	**0.05**
	PCOS *	0.363	[−0.274;1.043]	1.049	415.224	0.263
	Contraceptive *	−0.709	[−1.507;0.088]	−1.761	122.556	**0.081**
	Pregnancy Concerns	−1.303	[−2.264;−0.342]	−2.665	523	**0.008**
	Breast Health	−0.355	[−1.233;0.524]	−0.793	523	0.428
	Menopause Symptoms	−0.731	[−1.760;0.298]	−1.395	523	**0.164**
	Other gynecological conditions	−0.439	[−1.264;0.387]	−1.044	523	0.297

*Variances are not equally assumed

**Table 8:** Age differences by drivers for AI use

Variable	Grouping Variable	Mean Difference	95% CI	t	df	*p* value
Age (years)	Save Money *	0.721	[0.089;1.370]	2.243	499.293	**0.025**
	Save Time *	0.488	[−0.263;1.239]	1.279	248.317	**0.202**
	Embarrassment *	0.926	[0.301;1.551]	2.911	521.418	**0.004**
	Access to healthcare	0.407	[−0.319;1.133]	1.102	523	0.271
	Fear of being judged*	1.169	[0.545;1.792]	3.685	427.329	**< 0.001**

### Multivariate analysis

A multiple linear regression model was used to identify predictors of age in relation to AI chatbot engagement. The model explained 10.1% of the variance in age (Table 9).

**Table 9:** Multivariate predictors of age *Only significant results are included*

Predictor	B (Unstandardized)	Beta (Standardized)	*p*-value
Pregnancy Concerns (AI Use)	1.443	0.128	0.032
Health-related Field	1.039	0.138	0.001
Chronic Medications	1.185	0.107	0.012
Smoking	1.555	0.165	< 0.001
Fear of Being Judged	−0.972	−0.123	0.021

Five variables were independently associated with age. Participants who reported using AI for pregnancy-related concerns were significantly older (B = 1.443, *p* = 0.032), as were those working or studying in a health-related field (B = 1.039, *p* = 0.001), those taking chronic medications (B = 1.185, *p* = 0.012), and those who reported smoking (B = 1.555, *p* < 0.001). In contrast, reporting “fear of being judged” as a reason for using AI was associated with younger age (B = − 0.972, *p* = 0.021).

### Cluster analysis

To explore patterns of chatbot engagement, we conducted a K-means cluster analysis which identified three distinct user profiles based on their chatbot usage across health topics (Table 10):

**Table 10:** Cluster-Based AI use patterns (% reporting use)

Topic	Cluster 0	Cluster 1	Cluster 2
Mental Health	74.2%	26.1%	87.5%
Physical Fitness	84.4%	39.2%	89.6%
Menstrual Problems	75.8%	43.3%	91.7%
Pregnancy Concerns	72.6%	26.5%	89.6%
PCOS	71.5%	36.1%	85.4%
Performance Enhancing Drugs	12.4%	1.0%	41.7%
Breast Health	11.3%	2.4%	16.7%

- Cluster 0 (35.4%): Moderate to high use across both general and intimate health topics.
- Cluster 1 (55.4%): Low-engagement users with minimal AI reliance.
- Cluster 2 (9.1%): Super-users with the highest engagement, particularly for sensitive and stigmatized health issues.

Notably, users with the highest engagement (Cluster 2) reported the highest usage rates for mental health (87.5%), physical fitness (89.6%), menstrual problems (91.7%), and pregnancy concerns (89.6%). These users were also far more likely than others to consult AI for PCOS (85.4%), and even performance-enhancing drugs (41.7%).

## Discussion

This study sheds light on how young Lebanese women are increasingly turning to large language models as discreet confidants for gynecological and intimate health concerns. In a sociocultural context where women’s health needs are often met with silence and stigma [10, 12], our findings suggest that AI chatbots serve as accessible, anonymous, and nonjudgmental spaces for health-related inquiries. These tools are not merely digital novelties but are becoming practical alternatives for addressing culturally sensitive topics, particularly among younger users [15].

By examining the factors that increase or decrease usage, along with age-based engagement patterns, this study places LLM use at the intersection of technological acceptance, stigma navigation, and health-seeking behavior. Nearly half of participants reported consulting chatbots for menstrual issues, with considerable engagement for concerns like PCOS and vaginal infections. These figures denote the growing reliance on LLMs for gynecological concerns that are often masked in cultural taboos across the MENA region [10, 11]. The preference for AI in navigating such topics aligns with the notion that chatbots, by offering anonymity and neutrality, can reduce barriers like shame and fear of judgment in discussing sensitive health issues [6, 42], thereby facilitating access to information that might otherwise be difficult to obtain due to societal constraints. This echoes Marcantonio et al.‘s conclusion that AI platforms offer a “judgment-free zone” for taboo health topics [6].

### Barriers and drivers among younger women

Despite this enthusiastic uptake, participants expressed considerable reservations. The predominant barriers were accuracy concerns and the lack of physical examination, reflecting a tension between AI’s perceived utility and its limitations in replicating the depth of clinical expertise. Moreover, trust scores in AI were moderate, reflecting the caution young users exercise despite the convenience of these tools. Similarly, Palanica et al. demonstrated that physicians remain cautious about the diagnostic accuracy and safety of chatbots, particularly when physical assessments are necessary [43]. Furthermore, the absence of face-to-face interaction contributes to a diminished sense of trust and connection, which are often crucial when seeking care for intimate health issues. These concerns align with broader research about AI technologies, concluding that they should be viewed as complementary aids rather than replacements for healthcare professionals, emphasizing the ongoing need for human oversight and personal interaction in clinical settings [43–46].

On the other hand, the primary drivers were avoiding embarrassment and judgmental critiques, notably among younger adults, as well as saving time, suggesting that participants valued LLMs for their immediacy and discretion. Qualitative research supports these findings by highlighting the ways in which chatbots provide a safe and judgment-free space for discussing stigmatized health issues. Nadarzynski et al. found that although chatbots were generally less preferred than in-person consultations, many participants, particularly young people, valued them for their anonymity and privacy when disclosing sensitive sexual and reproductive health concerns [44]. Similarly, Branley-Bell et al. reported that chatbots create a “safe psychological space” that reduces embarrassment and encourages disclosure of taboo symptoms [47]. Additionally, younger populations are known to be particularly sensitive to stigma and fear of judgment when seeking health advice, which further explains their preference for discreet digital tools [48]. These insights underline how, beyond quantitative patterns, qualitative evidence reveals the psychological mechanisms that may explain young women’s willingness to engage with AI chatbots for intimate health concerns.

### Age and Topic-Specific patterns

Age differences were significant for several health topics and motivational drivers in this study, reflecting established patterns in health beliefs and behaviors across the lifespan [49]. Younger women’s preference for AI support with mental health, fitness, and menstrual issues aligns with evidence that this group faces unique psychosocial and physiological challenges [48]. In the context of mental health, this aligns with findings by Gulliver et al., who reported that younger individuals are more inclined to seek support through digital tools, particularly for stigmatized or sensitive issues [48]. Similarly, the preference for fitness-related chatbot use supports patterns observed by Gabarron et al., who found that AI-driven interventions aimed at increasing physical activity are especially well received among younger users [50]. Additionally, the association between younger age and cost-saving motivations in using AI chatbots aligns with the economic constraints faced by younger non-working students, who often lack stable financial resources and seek low-cost alternatives to traditional healthcare. Financial hardship among university students significantly influences their health-seeking behaviors, driving them toward more affordable, accessible digital health options [51].

Conversely, older participants’ increased use of chatbots for pregnancy-related queries correlates with the typical reproductive life course and shifting health priorities, as older university students demonstrate greater awareness and concern regarding pregnancy topics [52]. This contrasts with younger students, who predominantly engaged with chatbots for menstrual problems and mental health issues. These differences suggest that reproductive life stage and shifting health priorities strongly influence the topics for which women rely on digital tools, with older participants using chatbots for sensitive concerns that carry greater social stigma [53]. Additionally, besides biological and social transitions, this may reflect a life course-driven increase in pregnancy awareness, preparedness, and anxiety. Supporting this, Uyanik et al. found that higher maternal health literacy during pregnancy was paradoxically associated with increased pregnancy-related anxiety despite lower overall risk perception, indicating that greater awareness may increase concern and prompt information-seeking behavior, likely digital, that offers immediacy and discretion [54].

#### Clustered profiles

While the cluster analysis reveals distinct patterns of chatbot engagement, the interpretation of these user profiles shall be regarded as inferential and grounded solely in quantitative interaction data. The analysis identified three user profiles: a majority with low overall engagement, a group with moderate to high engagement across topics, and a small group of highly engaged users focused on sensitive and stigmatized health issues. This typology reflects the diverse ways in which users navigate AI chatbot technology, revealing meaningful variability in both frequency and depth of interaction [55].

The moderate to high engagement clusters align with prior findings showing that many young women are increasingly comfortable using AI tools for both general and intimate health matters, suggesting an emerging norm toward digital health acceptance, at least where the stakes are perceived to be relatively low [6, 7].

On the other hand, the predominance of low-engagement users signals that the traditional concerns did not disappear with the emergence of novel digital interfaces. Privacy apprehensions [22, 56] and the indispensable role of physical examination and human rapport [43–46], remain important obstacles to adoption, particularly in cases where clinical reassurance is as important as information.

Meanwhile, the super-user group provides a compelling contrast. This cluster, who engage extensively with LLMs across multiple domains, including mental health, fitness, menstrual concerns, and pregnancy, uses these tools not as novelties, but as necessities. Their behavior reflects a pragmatic and rational approach to healthcare, particularly in contexts where stigma or systemic barriers discourage conventional consultations. Existing literature supports this pattern, showing that individuals confronting sociocultural taboos often turn to anonymous digital health tools as a safe haven from judgment [6, 7, 44]. In other words, this subgroup demonstrates that, while chatbots may not replace doctors anytime soon, they are already filling a critical gap where silence, shame, or inaccessibility once prevailed [47, 57].

### Clinical implications

Findings from this study suggest that AI chatbots may be considered as part of a broader strategy to improve access to gynecological and intimate health support for young women, particularly in settings marked by stigma and healthcare limitations.

Healthcare providers might consider acknowledging the role these technologies play in facilitating early engagement, especially for individuals discouraged by embarrassment, social judgment, or financial barriers. Clinicians are encouraged to screen for digital health use during consultations, especially when discussing sensitive topics, to better understand patients’ information-seeking behaviors and potential misconceptions. Also, integrating AI tools into health education programs as well as collaborating with psychologists, digital health experts, and reproductive health specialists can support more comprehensive care. Integration of large language models and digital health into the global primary care framework and public health policies in Lebanon may offer advancements in patient support and protection. However, it is essential to maintain careful oversight of the information provided, not only to improve access and reduce neglect but also to remind patients that, like search engines such as Google, LLMs can possibly deliver misleading or inaccurate information.

In Lebanon, where cultural taboos and systemic healthcare gaps intersect, addressing both psychological and informational needs is crucial. Encouraging responsible use of AI while promoting health literacy can help reduce the risks associated with misinformation and can contribute to more informed decision-making. Targeted interventions that combine digital health resources with clinical oversight may serve to bridge gaps in access and trust, especially among university-aged women. Further research is necessary to assess long-term outcomes of chatbot engagement on health behaviors and to guide future clinical practices that reflect evolving patient preferences in digital health environments.

### Limitations

This study has several limitations. Its cross-sectional design precludes any conclusions about causality, as is common with this methodology. The use of snowball sampling, as participants were recruited through existing networks, may have generated greater sample homogeneity, leading to an overrepresentation of students who are more digitally connected and tech-savvy, while underrepresenting those with limited access to or lower familiarity with digital tools, thereby restricting the generalizability of the findings. Information bias is also a concern, since participants might have over- or underestimated their use of AI tools or the extent of their health concerns. Furthermore, although the questionnaire was pilot tested and refined accordingly, it was not derived from previously and cross-culturally validated instruments, which limits comparability with other studies and may affect measurement validity. Nonetheless, the pilot testing process improved the instrument’s clarity and appropriateness for the target population. In addition, the study relied exclusively on quantitative methods, which restricted our ability to explore in depth why participants perceive or trust AI chatbots in particular ways. As such, our interpretation of user profiles shall be considered exploratory and hypothesis-generating rather than definitive. Future research with a mixed-methods approach, including in-depth interviews or open-ended survey responses, would be essential to validate and enrich these findings.

## Conclusion

In conclusion, artificial intelligence chatbots hold significant potential as accessible, discreet tools for supporting both general and intimate health needs among young Lebanese women, particularly in a sociocultural context shaped by stigma and systemic healthcare constraints. Their capacity to offer nonjudgmental, on-demand information may be especially valuable for individuals facing barriers to traditional care. However, these technologies are not a substitute for the clinical judgment and relational depth that define human-centered care. Maximizing their value will require not only technological innovation but also thoughtful integration into broader health systems, to ensure that artificial intelligence complements, rather than replaces, the expertise and empathy of healthcare professionals.

## Supplementary Information

Supplementary material 1.

## Data Availability

Data is provided within the manuscript.

## References

[1] Bugaj TJ, Schwarz TA, Friederich H-C, Nikendei C. The curious physician: exploring the role of curiosity in professionalism, patient care, and well-being. Ann Med. 2024;56(1):2392887. 10.1080/07853890.2024.2392887. 39155851 10.1080/07853890.2024.2392887 PMC11334747

[2] De Angelis L, et al. ChatGPT and the rise of large Language models: the new AI-driven infodemic threat in public health. Front Public Health. Apr. 2023;11:1166120. 10.3389/fpubh.2023.1166120. 10.3389/fpubh.2023.1166120 PMC10166793 37181697

[3] Fattah FH. Comparative analysis of ChatGPT and Gemini (Bard) in medical inquiry: a scoping review. Front Digit Health. 2025;7:1482712. 10.3389/fdgth.2025.1482712. 39963119 10.3389/fdgth.2025.1482712 PMC11830737

[4] Ran B, Wang P, Liu L. Unlocking the potential of ChatGPT in male sexual health and dysfunction: a hold endeavor and comprehensive study. World J Mens Health. 2025;43(2):448–51. 10.5534/wjmh.240228. 39536769 10.5534/wjmh.240228 PMC11937348

[5] Hristidis V, Ruggiano N, Brown EL, Ganta SRR, Stewart S. ChatGPT vs Google for queries related to dementia and other cognitive decline: comparison of results. J Med Internet Res. 2023;25:e48966. 10.2196/48966. 37490317 10.2196/48966 PMC10410383

[6] Marcantonio T, et al. Hey ChatGPT, let’s talk about sexual consent. J Sex Res. 2023. 10.1080/00224499.2023.2254772. 37707442 10.1080/00224499.2023.2254772 PMC10937333

[7] Esmaeilzadeh P, Maddah M, Mirzaei T. Using AI chatbots (e.g., CHATGPT) in seeking health-related information online: the case of a common ailment. Comput Hum Behav Artif Hum. 2025;3:100127. 10.1016/j.chbah.2025.100127.

[8] Ilkkaracan P. Commentary: Sexual health and human rights in the Middle East and North Africa: progress or backlash? Glob Public Health. 2015;10(2):268–70. 10.1080/17441692.2014.986173. 25585195 10.1080/17441692.2014.986173 PMC4318083

[9] Zaki SA, et al. Prevalence of STIs, sexual practices and substance use among 2083 sexually active unmarried women in Lebanon. Sci Rep. 2021;11(1):9855. 10.1038/s41598-021-89258-5. 33972604 10.1038/s41598-021-89258-5 PMC8110545

[10] Mohd NF, Tohit, Haque M. Forbidden conversations: A comprehensive exploration of taboos in sexual and reproductive health. Cureus, 16, 8, p. e66723, 10.7759/cureus.66723 10.7759/cureus.66723 PMC11319820 39139803

[11] Oraby D. Sexuality education for youth and adolescents in the middle East and North Africa region: a window of opportunity. Glob Health Sci Pract. 2024;12(1):e2300282. 10.9745/GHSP-D-23-00282. 38290752 10.9745/GHSP-D-23-00282 PMC10906548

[12] Hamdanieh M, et al. Assessment of sexual and reproductive health knowledge and awareness among single unmarried women living in Lebanon: a cross-sectional study. Reprod Health. 2021;18:24. 10.1186/s12978-021-01079-x. 33509225 10.1186/s12978-021-01079-x PMC7842035

[13] Li SW, et al. ChatGPT outscored human candidates in a virtual objective structured clinical examination in obstetrics and gynecology. Am J Obstet Gynecol. 2023;229(2):172.e1-172.e12. 10.1016/j.ajog.2023.04.020. 37088277 10.1016/j.ajog.2023.04.020

[14] Shahsavar Y, Choudhury A. User intentions to use ChatGPT for self-diagnosis and health-related purposes: cross-sectional survey study. JMIR Hum Factors. 2023;10:e47564. 10.2196/47564. 37195756 10.2196/47564 PMC10233444

[15] Kim H-K. The effects of artificial intelligence chatbots on women’s health: a systematic review and meta-analysis. Healthcare. 2024;12(5):534. 10.3390/healthcare12050534. 38470645 10.3390/healthcare12050534 PMC10930454

[16] Holen ÅS, Martiniussen MA, Bergan MB, Moshina N, Hovda T, Hofvind S. Women’s attitudes and perspectives on the use of artificial intelligence in the assessment of screening mammograms. Eur J Radiol. 2024;175:111431. 10.1016/j.ejrad.2024.111431. 38520804 10.1016/j.ejrad.2024.111431

[17] Young AT, Amara D, Bhattacharya A, Wei ML. Patient and general public attitudes towards clinical artificial intelligence: a mixed methods systematic review. Lancet Digit Health. Sept. 2021;3(9):e599–611. 10.1016/S2589-7500(21)00132-1. 10.1016/S2589-7500(21)00132-1 34446266

[18] Khullar D, Casalino LP, Qian Y, Lu Y, Krumholz HM, Aneja S. Perspectives of patients about artificial intelligence in health care. JAMA Netw Open. May 2022;5(5):e2210309. 10.1001/jamanetworkopen.2022.10309. 10.1001/jamanetworkopen.2022.10309 PMC9069257 35507346

[19] Thirunavukarasu AJ, Ting DSJ, Elangovan K, Gutierrez L, Tan TF, Ting DSW. Large language models in medicine. Nat Med. 2023;29(8):1930–40. 10.1038/s41591-023-02448-8. 37460753 10.1038/s41591-023-02448-8

[20] Wang L, et al. Applications and concerns of ChatGPT and other conversational large language models in health care: systematic review. J Med Internet Res. 2024;26:e22769. 10.2196/22769. 39509695 10.2196/22769 PMC11582494

[21] Mu Y, He D. The potential applications and challenges of ChatGPT in the medical field. Int J Gen Med. 2024;17:817–26. 10.2147/IJGM.S456659. 38476626 10.2147/IJGM.S456659 PMC10929156

[22] Wang C, Liu S, Yang H, Guo J, Wu Y, Liu J. Ethical considerations of using ChatGPT in health care. J Med Internet Res. 2023;25:e48009. 10.2196/48009. 37566454 10.2196/48009 PMC10457697

[23] Montazeri M, Galavi Z, Ahmadian L. What are the applications of ChatGPT in healthcare: gain or loss? Health Sci Rep. 2024;7(2):e1878. 10.1002/hsr2.1878. 38361810 10.1002/hsr2.1878 PMC10867364

[24] Kuang Y-R, Zou M-X, Niu H-Q, Zheng B-Y, Zhang T-L, Zheng B-W. ChatGPT encounters multiple opportunities and challenges in neurosurgery. Int J Surg. 2023;109(10):2886–91. 10.1097/JS9.0000000000000571. 37352529 10.1097/JS9.0000000000000571 PMC10583932

[25] Hackl E. Assessing ChatGPT’s use of person-first language in healthcare conversations, *Discov. Artif. Intell.*, vol. 4, no. 1, pp. 1–10, Dec. 2024, 10.1007/s44163-023-00099-9

[26] Armbruster J, Bussmann F, Rothhaas C, Titze N, Grützner PA, Freischmidt H. Doctor ChatGPT, can you help me?’ the patient’s perspective: cross-sectional study. J Med Internet Res. 2024;26:e58831. 10.2196/58831. 39352738 10.2196/58831 PMC11480680

[27] Daher M, Koa J, Boufadel P, Singh J, Fares MY, Abboud JA. Breaking barriers: can ChatGPT compete with a shoulder and elbow specialist in diagnosis and management? JSES Int. 2023;7(6):2534–41. 10.1016/j.jseint.2023.07.018. 37969495 10.1016/j.jseint.2023.07.018 PMC10638599

[28] Bou Sanayeh E, Chamieh CE. The fragile healthcare system in Lebanon: sounding the alarm about its possible collapse. Health Econ Rev. 2023;13:21. 10.1186/s13561-023-00435-w. 37014485 10.1186/s13561-023-00435-w PMC10071460

[29] Fleifel M, Abi Farraj K. The Lebanese healthcare crisis: an infinite calamity. Cureus, 14, 5, p. e25367, 10.7759/cureus.25367 10.7759/cureus.25367 PMC9235031 35769680

[30] Farran N. Mental health in Lebanon: tomorrow’s silent epidemic. Mental Health & Prevention. 2021;24:200218. 10.1016/j.mhp.2021.200218. 34660191 10.1016/j.mhp.2021.200218 PMC8503814

[31] Itani R. A crisis amidst many others: COVID-19 response satisfaction during the economic collapse and post-Beirut port explosion in Lebanon. J Public Health Dev. 2024;22(1):1. 10.55131/jphd/2024/220102.

[32] Shallal A, Lahoud C, Zervos M, Matar M. Lebanon is losing its front line. J Glob Health. 2021;11:03052. 10.7189/jogh.11.03052. 33828836 10.7189/jogh.11.03052 PMC8005300

[33] Clark KA, Keene,Danya E, Pachankis, John E, Fattal, Omar, Rizk, Nesrine, and K. and, Khoshnood. A qualitative analysis of multi-level barriers to HIV testing among women in Lebanon, *Cult. Health Sex.*, vol. 19, no. 9, pp. 996–1010, Sept. 2017, 10.1080/13691058.2017.1282045 10.1080/13691058.2017.1282045 28276925

[34] Liu F, Chang X, Zhu Q, Huang Y, Li Y, Wang H. Assessing clinical medicine students’ acceptance of large language model: based on technology acceptance model. BMC Med Educ. 2024;24:1251. 10.1186/s12909-024-06232-1. 39490999 10.1186/s12909-024-06232-1 PMC11533422

[35] Holden RJ, Karsh B-T. The technology acceptance model: its past and its future in health care. J Biomed Inf. 2010;43(1):159–72. 10.1016/j.jbi.2009.07.002. 10.1016/j.jbi.2009.07.002 PMC2814963 19615467

[36] Cohen J. Statistical power analysis for the behavioral sciences. 2nd ed. New York: Routledge; 2013. 10.4324/9780203771587.

[37] Latt PM, et al. Evaluation of artificial intelligence (AI) chatbots for providing sexual health information: a consensus study using real-world clinical queries. BMC Public Health. 2025;25(1):1788. 10.1186/s12889-025-22933-8. 40375254 10.1186/s12889-025-22933-8 PMC12080182

[38] Mills R, Mangone ER, Lesh N, Mohan D, Baraitser P. Chatbots to improve sexual and reproductive health: realist synthesis. J Med Internet Res. 2023;25:e46761. 10.2196/46761. 37556194 10.2196/46761 PMC10448286

[39] Inkster B, Kadaba M, Subramanian V. Understanding the impact of an AI-enabled conversational agent mobile app on users’ mental health and wellbeing with a self-reported maternal event: a mixed method real-world data mHealth study. Front Glob Womens Health. 2023;4:1084302. 10.3389/fgwh.2023.1084302. 37332481 10.3389/fgwh.2023.1084302 PMC10272556

[40] Hosmer DW and S. and, Lemesbow. Goodness of fit tests for the multiple logistic regression model, *Commun. Stat. - Theory Methods*, vol. 9, no. 10, pp. 1043–1069, Jan. 1980, 10.1080/03610928008827941

[41] Jain AK. Data clustering: 50 years beyond K-means. Pattern Recogn Lett. 2010;31(8):651–66. 10.1016/j.patrec.2009.09.011.

[42] Basu K, Sinha R, Ong A, Basu T. Artificial intelligence: how is it changing medical sciences and its future? Indian J Dermatol. 2020;65(5):365–70. 10.4103/ijd.IJD_421_20. 33165420 10.4103/ijd.IJD_421_20 PMC7640807

[43] Palanica A, Flaschner P, Thommandram A, Li M, Fossat Y. Physicians’ perceptions of chatbots in health care: cross-sectional web-based survey. J Med Internet Res. 2019;21(4):e12887. 10.2196/12887. 30950796 10.2196/12887 PMC6473203

[44] Nadarzynski T, Miles O, Cowie A, Ridge D. Acceptability of artificial intelligence (AI)-led chatbot services in healthcare: a mixed-methods study. Digit Health. 2019;5:2055207619871808. 10.1177/2055207619871808. 31467682 10.1177/2055207619871808 PMC6704417

[45] Reis F, Lenz C. Performance of artificial intelligence (AI)-Powered chatbots in the assessment of medical case reports: qualitative insights from simulated scenarios. Cureus, 16, 2, p. e53899, 10.7759/cureus.53899 10.7759/cureus.53899 PMC10925004 38465163

[46] Altamimi I, Altamimi A, Alhumimidi AS, Altamimi A, Temsah M-H. Artificial intelligence (AI) chatbots in medicine: A Supplement, not a substitute. Cureus, 15, 6, p. e40922, 10.7759/cureus.40922 10.7759/cureus.40922 PMC10367431 37496532

[47] Branley-Bell D, Brown R, Coventry L, Sillence E. Chatbots for embarrassing and stigmatizing conditions: could chatbots encourage users to seek medical advice? Front Commun. 2023. 10.3389/fcomm.2023.1275127.

[48] Gulliver A, Griffiths KM, Christensen H. Perceived barriers and facilitators to mental health help-seeking in young people: a systematic review. BMC Psychiatry. 2010;10(1):113. 10.1186/1471-244X-10-113. 21192795 10.1186/1471-244X-10-113 PMC3022639

[49] Furnham A, Kirkcaldy B. Age and sex differences in health beliefs and behaviours. Psychol Rep. 1997;80(1):63–6. 10.2466/pr0.1997.80.1.63. 9122353 10.2466/pr0.1997.80.1.63

[50] Gabarron E, Larbi D, Rivera-Romero O, Denecke K. Human factors in AI-driven digital solutions for increasing physical activity: scoping review. JMIR Hum Factors. 2024;11:e55964. 10.2196/55964. 38959064 10.2196/55964 PMC11255529

[51] Zhang J, et al. Young adult perspectives on artificial intelligence–based medication counseling in China: discrete choice experiment. J Med Internet Res. 2025;27:e67744. 10.2196/67744. 40203305 10.2196/67744 PMC12018864

[52] San Lazaro Campillo I, Meaney S, Sheehan J, Rice R, O’Donoghue K. University students’ awareness of causes and risk factors of miscarriage: a cross-sectional study. BMC Womens Health. 2018;18:188. 10.1186/s12905-018-0682-1. 30453933 10.1186/s12905-018-0682-1 PMC6245715

[53] Miles O, West R, Nadarzynski T. Health chatbots acceptability moderated by perceived stigma and severity: a cross-sectional survey. Digit Health. 2021;7:20552076211063012. 10.1177/20552076211063012. 34917391 10.1177/20552076211063012 PMC8670785

[54] Uyanik A, Koç G, Ardiç M. The effect of pregnancy health literacy on risk perception in pregnancy and pregnancy anxiety. BMC Pregnancy Childbirth. 2025;25:664. 10.1186/s12884-025-07792-w. 40483496 10.1186/s12884-025-07792-w PMC12145611

[55] Yang Y, Tavares J, Oliveira T. A new research model for artificial intelligence-based well-being chatbot engagement: survey study. JMIR Hum Factors. 2024;11:e59908. 10.2196/59908. 39527812 10.2196/59908 PMC11589509

[56] Coghlan S, Leins K, Sheldrick S, Cheong M, Gooding P, D’Alfonso S. To chat or bot to chat: ethical issues with using chatbots in mental health. Digit Health. June 2023;9:20552076231183542. 10.1177/20552076231183542. 10.1177/20552076231183542 PMC10291862 37377565

[57] Aggarwal A, Tam CC, Wu D, Li X, Qiao S. Artificial Intelligence–Based chatbots for promoting health behavioral changes: systematic review. J Med Internet Res. Feb. 2023;25(1):e40789. 10.2196/40789. 10.2196/40789 PMC10007007 36826990

