# Supplementary material for: AI chatbots as ‘pocket doctors’: intimate health support for young women in Lebanon
Source: BMC Public Health. 2025 Nov 17;25:3989. doi: 10.1186/s12889-025-25386-1 (PMC12625598; doi:10.1186/s12889-025-25386-1)
Supplement: Supplementary file 1 — Supplementary material 1. [file 12889_2025_25386_MOESM1_ESM.pdf]

ChatGPT : Your friendly pocket Doctor

عزيزي المشارك،

هذه الدراسة تُجرى من قبل قسم جراحة المسالك البولية في جامعة الروح القدس في الكسليك ومستشفى سيدة المعونات الجامعي في جبيل، لبنان.
تهدف هذه الدراسة إلى تقييم الاعتماد في لبنان على AI (Artificial Intelligence) chatbots (ChatGPT, Gemini, Claude AI,...) للإجابة عن مخاوف تتعلق بصحتهم الحميمة.

نقدّر جدًا الوقت الذي تخصصه للإجابة على هذا الاستبيان، ونؤكد لك أن إجاباتك ستبقى مجهولة الهوية تمامًا.

لن يتم جمع أو تخزين أو مشاركة أي معلومات شخصية تحت أي ظرف من الظروف.

شكرًا لمساهمتك القيمة في هذه الدراسة.

,Dear Participant

This study is being conducted by the Department of Urology at Holy Spirit University of Kaslik .and Notre Dame des Secours University Hospital in Jbeil, Lebanon

The study aims to evaluate the reliance in Lebanon on AI (Artificial Intelligence) chatbots .(ChatGPT, Gemini, Claude AI, etc.) to address concerns related to their intimate health

We greatly appreciate the time you dedicate to answering this survey and assure you that your .responses will remain completely anonymous

.No personal information will be collected, stored, or shared under any circumstances

.Thank you for your valuable contribution to this study

* Indicates required question

1. Do we have your consent to continue with the questionnaire ? *

Mark only one oval.

- Yes
- No

Sociodemographic information

2. What is your age ? (Years) *

3. Where do you currently live ? *

Mark only one oval.

- Beirut
- Mount Lebanon
- Bequaa Valley
- South Lebanon
- North Lebanon

4. In which university are you currently enrolled ? *

Mark only one oval.

- Holy Spirit University of Kaslik - USEK
- Lebanese University
- American University of Beirut
- Lebanese American University
- Saint Joseph University
- University of Balamand
- St. George University of Beirut
- Notre Dame University
- Sagesse University
- Not enrolled in a university
- Other: ______________________

5. Do you work or are you currently studying a health-related field? *

Mark only one oval.

- Yes
- No

6. What is your main type of health coverage ? (if no health coverage please click on Out of Pocket expenses) *

Mark only one oval.

- Private Insurance
- Public Coverage
- Out of pocket expenses

7. Do you take any chronic medications *

Mark only one oval.

- Yes
- No

8. What is your current status *

Mark only one oval.

- Single
- In a relationship
- Married
- Widowed

9. Do you *

Mark only one oval per row.

	Yes	No
**Smoke**		
**Consume alcoholic beverages**		

10. How would you evaluate your personal financial burden overall? *

Mark only one oval.

	1	2	3	4	5	6	7	8	9	10	
No F	◯	◯	◯	◯	◯	◯	◯	◯	◯	◯	Overwhelming Financial Burden

Reliance on AI (Artificial Intelligence) chatbots for General Health Questions

11. Have you ever used AI for : *

Mark only one oval per row.

	Yes	No
**Mental health related questions (e.g., dealing with stress, anxiety, depression, or emotional well-being)?**	◯	◯
**Physical fitness related questions (e.g., muscle gain, workout plans, exercise routines, or fitness advice) ?**	◯	◯
**Performance enhancing drugs such as steroids?**	◯	◯
**Fitness Supplements (e.g., creatine, whey protein powder, Ashwagandha...)**	◯	◯
**Nutrition related questions (e.g., weight loss, diet plans, calorie tracking, or advice on healthy eating) ?**	◯	◯

12. On a scale of 1-10, how trustworthy do you find AI chatbots' health-related answers? *

Mark only one oval.

	1	2	3	4	5	6	7	8	9	10	
No t	◯	◯	◯	◯	◯	◯	◯	◯	◯	◯	Full trust

13. How accurate do you think AI is compared to medical professionals? *

Mark only one oval.

- ◯ Inferior to a doctor
- ◯ Equal to a doctor
- ◯ Superior to a doctor

14. Would you compare answers for a medical concern between your doctor and AI ? *

Mark only one oval.

- ◯ Yes
- ◯ No

Medical Conditions and Intimate Issues

15. Have you ever consulted AI for issues like *

Mark only one oval per row.

	Yes	No
**Menstrual problems (irregular cycles, painful or missed periods, heavy bleeding)**	◯	◯
**Sexually transmitted diseases (infections passed through sexual contact)**	◯	◯
**Infertility (trouble getting pregnant or having kids, concerns about fertility)**	◯	◯
**Vaginal Discharge and Infections (unusual discharge, itching, bad smell, or infections)**	◯	◯
**Urinary Tract Infections (burning, pain, or needing to pee often)**	◯	◯
**Pelvic Pain (constant aches, cramps** not from [illegible]	◯	◯

not from periods)		
Pain during sex, Vaginal tightness or dryness	◯	◯
Urinary Incontinence (leaking urine or losing control of urination)	◯	◯
Polycystic Ovary Syndrome (PCOS) (irregular periods, extra hair, acne, or weight struggles)	◯	◯
Contraceptive Options and Side Effects (choosing the right birth control method)	◯	◯
Pregnancy Concerns (symptoms, complications, or questions about prenatal care)	◯	◯
Breast Health (lumps, pain, or nipple discharge)	◯	◯
Menopause Symptoms (hot flashes, mood changes,	◯	◯

mood changes, vaginal dryness, or irregular cycles)		
Other Gynecologic Conditions (cancers, organs like the bladder or uterus dropping down, viruses, vaginal warts...)	◯	◯

16. Do you feel more comfortable using AI for intimate health issues over consulting a doctor? *

Mark only one oval.

- ◯ Yes
- ◯ No

17. Do you think using AI reduces the cultural stigma associated with asking questions about intimate health issues? *

Mark only one oval.

- ◯ Yes
- ◯ No

18. Do you believe AI can independently address health concerns, potentially reducing the need to consult a doctor? *

Mark only one oval.

- Yes
- No

19. Do you believe AI is a private and safe platform for sensitive health inquiries? *

Mark only one oval.

- Yes
- No

Attitudes and Reliance on AI chatbots

20. How likely are you to rely on each of the following *

Mark only one oval per row.

	1 (I don' t rely on this source at all)	2	3	4	5	6	7	8
**AI Chatbots (ChatGPT, Gemini,...)**								
**Google**								
**Social Media Platforms (Youtube, TikTok,...)**								
**Consulting a health professional**								

21. Why would you rely on AI for health advice *

Mark only one oval per row.

	Yes	No
**To save money**	⬭	⬭
**To save time**	⬭	⬭
**Embarrassment discussing issues in person**	⬭	⬭
**Lack of access to healthcare providers**	⬭	⬭
**Fear of being judged**	⬭	⬭

22. What prevents you from relying entirely on AI for health concerns? *

Mark only one oval per row.

	Yes, this prevents me from entirely relying on AI	No, this doesn't prevent me from entirely relying on AI
**Accuracy concerns and Fear of Missing Details**	⬭	⬭
**Lack of emotional support**	⬭	⬭
**Lacks contextual understanding**	⬭	⬭
**Need for physical examination**	⬭	⬭
**Privacy Risks**	⬭	⬭

This content is neither created nor endorsed by Google.

Google Forms
